# Dr. Garland’s Letter

**Published:** 1855-12

**Authors:** G. W. Garland

**Affiliations:** Lawrence


Dr. Garland's Letter. *Dr. Peaslee:* Dear Sir.—When I saw you last I forgot to call your attention to the medical properties and uses of the Yellow Jasmine, or Jassamine, or Gelseminum Semperverius, which grows wild in considerable abundance in Virginia. I think it will be classed among the nervines; still it is given in febrile diseases in from twelve to sixty drops, particularly in fever and ague, with three to five grains of quinine every two hours, till three doses are given. It affects some much more than others. Usually in twelve to twenty drop doses of the tincture a feeling is produced of *utter inability* to move, particularly the arms—still you *can* move. It is unlike anything ever felt. I took twenty-five drops sitting in my office, and when the operation came on I tried to think of some feeling to compare it with, and could think of nothing but the first dawn of consciousness after a full or complete syncope. It is a species of intoxication, which goes off leaving no headache or other unpleasant feeling.

In neuralgia, rheumatism, and many uterine diseases, it has been found very efficacious. I have given the tincture in several cases in neuralgia with flattering results. The greatest benefit will be realized in the *periodical* type of that disease.

Have the goodness to call the attention of the Society at Concord to this medicine. Some one may know something more of its properties and uses. The bark of the root is the part used. Some prefer to use it with cornine instead of quinine—cornine, from Cornus Florida. When better understood, it will be a remedy of great value and efficacy, I have no doubt.

Respectfully, yours,

Lawrence, June 1, 1855. G. W. GARLAND.

[*Transactions N. H. Medical Society.*

